# Navigating the Complexities Involving the Identification of Botulinum Neurotoxins (BoNTs) and the Taxonomy of BoNT-Producing Clostridia

**DOI:** 10.3390/toxins15090545

**Published:** 2023-09-03

**Authors:** Theresa J. Smith, Kristin M. Schill, Charles H. D. Williamson

**Affiliations:** 1 Pathogen and Microbiome Institute, Northern Arizona University, Flagstaff, AZ 86011, USA; chase.williamson@nau.edu; 2 Food Research Institute, University of Wisconsin-Madison, Madison, WI 53706, USA; kristin.schill@wisc.edu

**Keywords:** botulinum neurotoxins, *Clostridium*, nomenclature, taxonomy

## Abstract

Botulinum neurotoxins are a varied group of protein toxins that share similar structures and modes of activity. They include at least seven serotypes and over forty subtypes that are produced by seven different clostridial species. These bacterial species are not limited strictly to BoNT-producers as neuro-toxigenic and non-neuro-toxigenic members have been identified within each species. The nomenclature surrounding these toxins and associated bacteria has been evolving as new isolations and discoveries have arisen, resulting in challenges in diagnostic reporting, epidemiology and food safety studies, and in the application of therapeutic products. An understanding of the intricacies regarding the nomenclature of BoNTs and BoNT-producing clostridia is crucial for communication that allows for accurate reporting of information that is pertinent to each situation.

## 1. History Involving Discovery of BoNTs and BoNT-Producing Clostridia

Describing the diversity within botulinum neurotoxins (BoNTs) and the bacteria that produce them is challenging. Just when a workable nomenclature system is proposed, novel organisms and toxins are discovered that do not fit the system. Changes in how toxins and bacteria are identified due to the shift from the use of serology and physiological characteristics for identification to genetic/genomic methods has also impacted how we report and categorize these entities.

The idea that botulism was caused by a single toxin moiety produced by a particular anaerobic bacterium [1] was rapidly disabused when subsequent BoNT-producing bacteria were isolated that: (a) differed from the initial bacterium described by Van Ermengem in basic metabolic characteristics [2], and (b) produced toxins that were serologically distinct from the toxin that was examined by Van Ermengem [3]. These toxins were designated toxin type A and toxin type B based on serological studies [4]. Subsequently, five additional serologically distinct toxins (C–G) were identified that have been associated with seven bacterial species [5]. The latest identifications of novel botulinum or botulinum-like toxins have been via genomic mining techniques, so the use of the term “serotype” is gradually changing to “toxinotype” or reverting to “toxin type”.

Throughout the past century, multiple changes in bacterial genus and species designations have been observed, and neurotoxin nomenclature has been expanded from simply “botulinum toxin” to encompass identifications of serotypes, toxinotypes, subtypes, and novel BoNT-like toxins. Table 1 contains a historical outline of these discoveries and changes.

As isolation and identification methods for these clostridia advanced, a wide array of toxins and toxin-producing bacteria were discovered. Similar complexities have been noted in other clostridial species, such as *C. perfringens*, that may produce at least eight major toxins (alpha, beta, epsilon, iota, enterotoxin, Beta2, TpeL, and NetB) [31], and *C. novyi*, that may produce at least four major toxins (alpha, beta, delta, epsilon) [32]. However, one distinguishing characteristic of the botulinum neurotoxins is their production by multiple clostridial species.

While botulinum neurotoxins retain similar tertiary structures and produce comparable clinical signs and symptoms in humans and susceptible animals, they significantly differ in amino acid composition. As new toxin types were discovered, serotype level amino acid differences were shown to range from 37% to 70% [33], and it is these differences that are responsible for an inability of antiserum against one toxin serotype to neutralize other toxin serotypes. This serotype specificity not only impacts neurotoxin detection/diagnosis, but treatment options as well.

Further diversity has been identified within each toxin serotype. There are currently over 40 toxin subtypes that generally differ by at least 2.6% in amino acid residues [34]. These differences do not require the use of specific antisera for detection, but differences in identification and treatment effectiveness due to differing antibody potencies have been noted, particularly with BoNT/CD [9], BoNT/F7 [35], and BoNT/HA [24]. In addition, toxin variants have been identified. These variants may differ by only one or two amino acids, or they may display significant differences that approach 2% compared with other members of that subtype.

As more toxins and BoNT-producing species were described, it became clear that there is a lack of consistency between the toxin type produced and the bacterial species that produces it, which adds an additional level of complexity when describing these entities. A single species may produce various toxin types, and some toxin types are produced by multiple species. BoNT/B toxins may be associated with three clostridial species, BoNT/E with two species, and BoNT/F with three species (Table 2).

## 2. Complexities Involving Botulinum Neurotoxin Nomenclature

The advent of serotyping as a tool to identify different protein toxins enabled the discovery that there was more than one botulinum toxin type. Early serotyping efforts encompassed agglutination methods using antisera generated against washed and heated bacterial cultures, so results involved interactions with both bacterial antigens and toxin proteins [37]. Similar studies were conducted employing complement fixation methods [38]. While these methods could identify and separate pure BoNT/A and BoNT/B cultures into multiple subgroups, they could only be used after isolation of pure bacterial cultures.

To definitively identify botulinum toxin in samples, the mouse neutralization assay was developed using toxin-specific antisera. Results from this assay can be obtained within 24–96 h and provide information on both toxicity and toxin type. Some drawbacks with the use of this assay include the occasional need for isolation and enrichment of a bacterial entity prior to testing, and it is a bioassay that requires the use of animals, that, in addition to the ethical issues, carries a high level of variability associated with the results. Despite this, the mouse neutralization assay remained the assay of choice for toxin detection and serotyping throughout the 20th century [39], and it continues to be the gold standard assay for toxin detection in food samples [40] in some places.

In addition to the need for animals, the use of standard toxin neutralization testing for serotyping has several other issues. When testing directly from food or stool samples, nonspecific mouse deaths may occur, and when testing cultures from pure isolates, their supernatants may fail to produce deaths in toxin-only control mice due to low toxin production [40]. Cross-neutralizations by more than one antiserum (BoNT/C and BoNT/D; BoNT/E and BoNT/F, etc.) have also occurred with some serotypes due to regions of shared DNA sequence [40]. In addition, failures to determine the serotype have occurred when bivalent or trivalent BoNT-producing strains were involved [41,42].

For example, discrepancies involving the serotyping of certain BoNT/C isolates led to designations of Cα for the BoNT/C toxins from fly larva and chickens in the United States, which were identified by Dr. Bengtson [8], and Cβ for those associated with cattle botulism in Australia, identified by Dr. Seddon [10]. The Cα toxins were later discovered to be chimeric toxins with enzymatic domain DNA sequences that are identical to *bont/C*, translocation sequences that are similar to both *bont/C* and *bont/D*, and receptor domain sequences that are closely related to *bont/D* [22]. A counterpart to this was discovered with BoNT/D strains, known as BoNT/DC, which showed reverse identity with respect to *bont/C* genes (*bont/D* enzymatic domain, shared sequence in the translocation domain, receptor binding domain sequence similar to *bont/C*) [22]. These chimeric sequences are the result of recombination events that occurred between the *bont/C* and *bont/D* genes.

A similar finding involving a novel toxin type occurred when an inability to neutralize a suspected toxin with any of the known antitoxins led researchers to believe they had identified an eighth botulinum toxin serotype, which they designated type H [25]. However, subsequent tests using more concentrated antitoxins revealed that this novel toxin could be neutralized with an excess of anti-BoNT/A antibody [43]. Sequencing of this toxin gene revealed it was a chimera with an enzymatic domain that was related to that of *bont/F5* (87% identity) and a receptor binding domain that was closely related to *bont/A* (92% identity), causing some to designate it *bont/FA*. It is of interest that this light-chain gene and the *bont/F5* light-chain gene share only ~67% identity with the other *bont/F* subtype genes. If an entire *bont* gene showed this level of diversity, it would be considered a new toxinotype (H). However, since only the light chains are unique, this novel toxin is generally referred to as BoNT/HA, while BoNT/F5 has retained its original subtype designation. These are a few illustrations of the complexities that may be encountered when determining the appropriate identity of these toxins.

The discovery that some *C. botulinum* bacteria can produce more than one toxin type was accomplished through testing different dilutions of culture supernatants against multivalent antisera, in addition to single-serotype-specific antisera [39]. Multiple bivalent strains producing both BoNT/A and BoNT/B, BoNT/A and BoNT/F, and BoNT/B and BoNT/F, and a trivalent strain producing BoNT/A and two subtypes of BoNT/F, have been reported.

Over the past 20 years, toxin identification has become less dependent on animal neutralization studies to determine the serotype and more dependent on rapid laboratory assays such as ELISA, activity assays such as enzymatic activity-based mass spectrometry (Endo-pep MS), and genetics-based tests such as PCR assays or analysis of nucleotide sequences derived from cultures or directly from contaminated samples [44]. Animal welfare concerns and the time required to test for botulinum toxin using the mouse neutralization bioassay fueled the development of these alternate identification/serotyping methods. While the initial seven toxin types (A–G) were identified using serologic assays, newer toxin types, such as BoNT/HA and BoNT/X, and most toxin subtypes have been identified using genetic sequence analysis [25,30]. Guidance for describing BoNT subtypes has been previously discussed [34].

Searches of databases have uncovered several genetic sequences that resemble botulinum neurotoxin genes. These sequences may represent rudimentary precursors of botulinum neurotoxins and, as such, they are of great interest for evolutionary studies. However, the enzymatic activities of the proteins they encode are ineffective and nonspecific, so the designation “botulinum neurotoxins” may be unwarranted in these instances. One particular toxin gene sequence was discovered that encodes a potential second botulinum toxin (BoNT/X) within the BoNT/B2 strain 111 [30]. While this novel toxin is a promising candidate for a status as a botulinum neurotoxin, whether it should be designated as such is currently undecided.

## 3. Nomenclature and Taxonomic Issues Surrounding BoNT-Producing *Clostridium* Species

Botulinum neurotoxins are unusual in that a variety of bacterial species contain *bont* genes and are capable of producing botulinum neurotoxins. Seven distinct clostridial species are known to produce BoNTs and are referred to here as: *C. parabotulinum*, *C. sporogenes*, *C. botulinum*, *C. novyi sensu lato*, *C. argentinense*, *C. butyricum*, and *C. baratii* (see Table 2 and [36]). The nomenclature surrounding these species has been modified numerous times over the past 125 years (Table 1). The initial designation for any bacteria causing botulism was *Bacillus botulinus* [1], denoting spore-forming, rod-shaped bacteria (*Bacillus*) that were typically isolated from sausages (*botulinus*). The genus name was changed in 1917 to separate the aerobic *Bacillus* species and the anaerobic *Clostridium* species, and to reflect the spindle shape of the bacterial rods during sporulation (*Clostridium*) [6]. A later publication separated the nomenclature into *Bacillus parabotulinus*/*Clostridium parabotulinum* for proteolytic organisms and *Bacillus botulinus/Clostridium botulinum* for nonproteolytic organisms [7]. However, the designation “*C. parabotulinum*” did not achieve widespread acceptance, and in 1953 it was proposed that any BoNT-producing organism be designated *C. botulinum* [12]. While this nomenclature was never officially recognized by the International Code of Nomenclature of Bacteria, the use of *Clostridium botulinum* to identify any BoNT-producing bacterium gained widespread acceptance over the latter half of the 20th century.

Some immediate issues with this nomenclature system included difficulties with *C. botulinum* bacteria that were identified as such prior to the loss of their *bont* genes and thus were no longer toxigenic [45,46], as well as isolates that were identified as *C. sporogenes* simply because they lacked toxin genes [36,37,47]. Further complications occurred with the discovery of toxigenic bacteria that were definitively not *C. botulinum*, such as BoNT/F-producing *C. baratii* [48], BoNT/E-producing *C. butyricum* [17], and the official species designation of BoNT/G-producing *C. argentinense* [19].

BoNT-producing bacteria have differing metabolic and physiological characteristics, which made species designations based solely on toxin production problematic. Some are proteolytic and some nonproteolytic, different bacterial groups have different growth temperature ranges and optimums, and importantly, the bacteria differ in their lipase and lecithinase reactions on egg yolk agar. These latter reactions were used to rapidly identify BoNT-producing bacteria [49], and failures to identify causative organisms have occurred when the expected reactions were not seen [17,50]. In order to counteract this problem, a system was developed that divided known BoNT-producing strains into four physiological groups based on variations in these characteristics (Table 3) [51].

While this grouping system was proven useful for identifying BoNT-producing bacteria with differing metabolic characteristics, it does not have legitimate standing in nomenclature. The International Code of Nomenclature of Bacteria states in its appendices, The term “group” is informal and has no nomenclatural standing. It may prove useful to designate informally a set of organisms having certain characteristics in common, provided that it is used with care and exact definition to avoid ambiguity. It should not be used to avoid the use of the correct name of a taxon such as genus or species” [54].

Further, as Collins et al. stated, “From a purely taxonomic viewpoint, it is recognized that a nomenclature based on BoNT production is unsatisfactory and that in most circumstances, the different metabolic groups of *Cl. botulinum* would be assigned to different species. In view of its utility to medical microbiologists, and in order to avoid possible confusion, however, there has been an understandable reluctance to initiate a major change in nomenclature” [52]. While there have been concerns about the use of legitimate species names to identify BoNT-producing bacterial species, such as *C. argentinense*, *C. baratii*, *C. butyricum*, and *C. sporogenes*, these bacteria have been routinely reported by their correct species names for over 30 years, with minimal to no confusion within the medical community.

**Figure 1:**
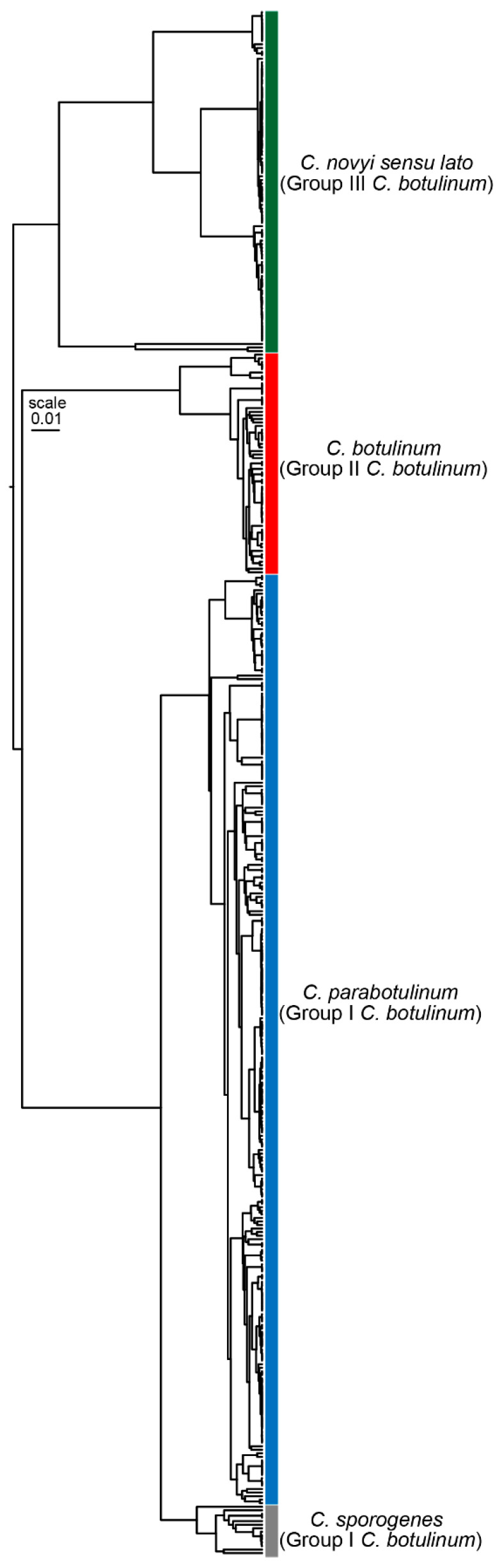
Hierarchical clustering of bacterial genome assemblies listed as “*Clostridium botulinum*” in the NCBI RefSeq database based on the average nucleotide identity. Average nucleotide identity values were calculated with pyani [58] using the MUMmer alignment option [59]. Species designations are labeled with colored bars. The close relationships between *C. parabotulinum* and *C. sporogenes* and diversity within each of the species are evident.

As stated by Dr. Prevot, there were predictions as early as 1966 that, “eventually bacterial classification will have an entirely genetic basis” [55]. These predictions were partly fulfilled with the genetic confirmations of the cases of botulism associated with *C. baratii* producing type F toxin and *C. butyricum* producing type E toxin [18], and the designation of BoNT/G-producing clostridia as *C. argentinense* [19] using DNA hybridization studies. Genetics-based classification methods progressed from DNA:DNA hybridization to analysis of 16S rRNA sequences to amplified fragment length polymorphism (AFLP), pulse-field gel electrophoresis (PFGE), multi-locus sequence typing (MLST), and whole-genome sequence analysis using average nucleotide identity (ANI) and single-nucleotide polymorphism (SNP) comparisons. The *C. botulinum* groups that were delineated through metabolic characteristics were confirmed as distinct species using these techniques [18,19,27,52,56]. Analysis of bacterial genome assemblies labeled “*C. botulinum*” in the NCBI RefSeq database shows the divergence of at least four species and reveals much diversity within each species designation (Figure 1, species are labeled as described in [36]). The close relationship between *C. parabotulinum* and *C. sporogenes* is obvious. In addition, several divergent lineages are evident within these species. Of particular note are the two lineages seen within *C. botulinum*, generally referred to as the E lineage and the BEF lineage [27,57], and the four lineages seen within *C. novyi sensu lato* [32]. Within all four of these species, a significant number of sub-lineages are seen, indicating the diversity present within each BoNT-producing species designation. An additional consideration is the inclusion of both neuro-toxigenic and non-neuro-toxigenic isolates in each of these categories.

There are multiple sources when considering the appropriate taxonomic and nomenclature status for BoNT-producing clostridial species, including the List of Bacterial Names in the International Code of Nomenclature of Bacteria [54], and Bergey’s Manual of Systematic Bacteriology [53]. However, these sources need updating in order to reflect the current taxonomic status of BoNT-producing clostridia [60,61].

## 4. Effective Communications Involving Botulism, Botulinum Neurotoxins, and BoNT-Producing Bacteria

The reporting of the identity of a particular toxin and the bacterial strain associated with it may vary depending on whether they are related to the diagnosis and treatment of human botulism cases, determination of causes of outbreaks in animals, epidemiology studies involving source tracking of human isolates or tracking of environmental samples, food protection studies, or identification of therapeutic toxin preparations. It is important to understand these differences in order to convey unambiguous information in each of these cases.

### 4.1. Diagnosis/Treatment of Human Botulism

Botulism is an intoxication that is caused by one of several related proteins (botulinum neurotoxins) that are produced by multiple species of clostridia. It is the presence of the toxin, not an infectious agent, that causes the neurological signs and symptoms that are characteristic of botulism. Thus, the isolation of an organism is not necessary for diagnosis or identifying treatment options.

Human botulism is suspected when patients present with clinical signs and symptoms that are associated with neurotoxicity, such as muscular weakness, double or blurred vision, slurred speech, and difficulty breathing (cdc.gov/botulism/symptoms.html; accessed on 26 May 2023). Confirmation is obtained through detection of botulinum neurotoxin in clinical samples [62]. This is accomplished using a variety of diagnostic methods, including the older mouse neutralization bioassay, ELISA assays, activity assays using mass spectrometry, and PCR assays.

Clinicians are primarily concerned with two things when they are considering a suspected botulism case: is it botulism, and what is the toxin type? The latter question becomes less important when treatment of adult botulism using heptavalent anti-botulinum antitoxin products is available [63]. However, the standard antitoxin treatment for infants (BabyBIG) is produced from serum obtained from human volunteers that are currently immunized with the bivalent anti-BoNT/AB vaccine, so this antitoxin is not effective against botulinum toxin types C–F, and thus knowledge of the toxin type is necessary when treating with BabyBIG [64].

Historical methods for determining toxin types were relatively slow and involved isolation of a toxigenic organism followed by mouse neutralization assays [62], but the information that is necessary for diagnosis and treatment of human botulism cases is now determined using rapid diagnostic methods. Currently, detection of *bont* genes directly from clinical or food samples using PCR is a widely used technique, as is detection of enzymatic activity by neurotoxins present within these samples (Endo-PEP assays) [44]. These types of assays are capable of providing doctors with the necessary information for treatment options and, as they are routinely used in larger laboratory settings or reference laboratories, same-day results are possible in many cases.

Based on the specific needs of clinicians, reporting of botulism cases could be cited as “botulinum neurotoxin type A identified” or, in the case of a positive culture, “botulinum neurotoxin-producing *Clostridium* species, type A” could be used, to avoid questions of toxigenicity and species ambiguity. These types of communications are already in use, as evidenced by multiple reports [65,66,67].

### 4.2. Animal Botulism

Botulism in animals poses a different set of challenges, as it is not commonly treated or proactively prevented through vaccination, except in areas where botulism is endemic. While human botulism cases are increasingly singular events or limited outbreaks affecting <10 persons (someone who consumes improperly home-canned foods, a person who injects contaminated heroin, or an infant that inhales a random spore from environmental dust), animal botulism can affect both domestic and wild animals, with catastrophic results. Animal botulism is often due to foodborne intoxication, but toxicoinfections have also been discovered. Some notable foodborne intoxications include an outbreak in 2004 that killed over 50,000 farmed foxes and minks within 1–2 days [68]. This outbreak was linked to macerated fish food contaminated with type C toxin. Another example that involved a natural outbreak occurred in 1996, where a massive bird die-off of approximately 15,000 fish-eating birds in the Salton Sea was traced back to dead/dying fish containing type C botulinum toxin [69]. Additional outbreaks in domestic animals have resulted in severe economic impacts to the cattle and poultry industries, and prevention through vaccination is not always possible or economically feasible [70,71]; therefore, limitation of existing outbreaks and prevention of future outbreaks is important [72].

Other sources for foodborne botulism include bacterial growth and toxin production in nature. It is known that spores from BoNT-producing organisms are ubiquitous in dry soils, wetland areas, and marine environments. A wide variety of animals, particularly migrating birds, may commonly carry spores in their intestinal tracts [71]. These spores remain dormant until the introduction of conditions conducive to germination, growth, and toxin production, such as anaerobiosis, appropriate ambient temperature, and a source of nutrients. Vegetation in stagnant water and dying animals can provide food sources for growth and toxin production of bacteria, and intoxications in susceptible animals may occur when they feed directly on these contaminated vegetation or sediments [73].

Other examples of intoxication cycles involve die-offs of lower food chain animals that provide incubation chambers for the growth and production of toxin in their carcasses. These carcasses are subsequently ingested by other animals that then succumb to botulism. For example, avian botulism may develop after the ingestion of flies/fly larva feeding on intoxicated carcasses [70,74] or following the ingestion of dead/dying intoxicated fish by fish-eating birds [75]. Similar occurrences are noted in domestically housed animals, where BoNT-producing bacteria germinate and produce toxin in food supplies for animals. Silage that is contaminated with dead animals provides a source of spores, a nutrient supply, and the necessary anaerobic conditions for the growth and production of BoNT-producing organisms [76]. Outbreaks due to contaminated silage may result in significant losses of animals, and they are often linked with the practice of supplementing cattle feed with broiler litter that may be contaminated with dead birds [77,78]. An additional intoxication cycle that is sometimes seen in ruminants suffering from phosphorus deficiency concerns the ingestion of bones from carcasses of intoxicated animals (lamsiekte, bulbar paralysis, pica), leading to deaths in cattle and sheep [76].

Toxicoinfections in animals do occur and they are reflective of human toxicoinfections, with syndromes that are similar to both human infant botulism (shaker foal syndrome [79]) and wound botulism [73].

Determination of the toxin type and subtype in animal botulism cases is important for both mitigation and prevention. While human botulism is associated with BoNT/A, /B, /E, and /F, animal botulism is typically caused by BoNT/C, BoNT/CD, BoNT/D, or BoNT/DC and BoNT/B. Different animals vary in their sensitivity to the toxin types, and animals that are resistant to botulinum toxins may harbor spores in their intestinal tracts that are unable to germinate until the animal expires, when their carcasses provide conditions for the bacteria to grow and produce the toxin [71]. These animals act as carriers that may spread spores during their lifetime and are a source of toxins to susceptible animals, and possibly humans [80], after death. Table 4 lists the susceptibilities of different animals to various toxin types.

### 4.3. Epidemiology/Environmental Studies

Epidemiology studies related to human or animal botulism are designed to trace the source of intoxications and prevent further spread of botulism when associated with foodborne outbreaks [81,82]. They may also be used to determine source batches of contaminated heroin related to wound botulism cases [83,84], and they have been employed in infant botulism cases as well [85,86]. The timing for reporting of results is not as critical as it is for diagnosis and treatment, and in these instances the preliminary reporting of the neurotoxin type is not always adequate to match isolates from multiple cases or clinical cases with potentially contaminated foods [87]. Here, it is necessary to establish whether multiple cases are caused by toxins that have been produced by the same organism. MLST typing is one method to establish relatedness [84], but definitive proof is obtained through comparisons of whole-genome sequences of organisms isolated from implicated foodstuffs with those from clinical samples of intoxicated patients [87,88,89].

Reporting of results often involves diagrammatic representations of phylogenetic data to illustrate relationships among various bacterial strains that are isolated from clinical samples and/or implicated causative agents in foodborne botulism [66,87,88]. Accurate species designations and toxin identification are also advisable for this type of research. These studies are not strictly related to human botulism cases, as similar methods are applied in animal botulism outbreaks and environmental sampling.

Environmental studies, in combination with source tracking of isolates related to human or animal cases, help to “map” areas where certain BoNT-producing bacteria may abound. For example, a study involving isolation of BoNT-producing organisms from soils along major east–west U.S. highways indicated a preponderance of type A strains in the United States west of 100° longitude, with majority of type B strains occurring east of the Mississippi River and above 36° north latitude [16]. These findings are echoed in the findings of infant botulism, which is more likely to be due to type A in the western U.S. and mostly due to type B in the mid-Atlantic states [90]. Additional studies show a predominance of type E foodborne botulism in Alaska and northern and western Canada that is linked with high levels of environmental isolates in these areas [91,92]. In Argentina, the prevalent subtype found in the soils is the BoNT/A2 subtype. Connections between the prevalent BoNT types in the environment and those associated with local infant botulism cases have been established in both the U.S. and Argentina [85,93,94], and this knowledge has been helpful with treatment choices.

The bacterial species that produces the neurotoxin can also provide information about conditions leading to botulism outbreaks. It is known that the classic nonproteolytic *C. botulinum* strains are psychotropic, and they are capable of growth and toxin production at near-freezing temperatures [95,96]. This explains the predominant isolation of nonproteolytic *C. botulinum* BoNT/E strains from human foodborne botulism cases in arctic and subarctic regions [90,97,98]. In addition, the differing habitats of proteolytic BoNT/A- and BoNT/B-producing strains (terrestrial) versus BoNT/F and BoNT/E producers (aquatic) is reflected in a predominant association of U.S. foodborne cases with vegetables and fruits in the former strains, and with fish/marine mammals as implicated foods in the latter.

Environmental studies play a part in epidemiology studies and animal botulism research; alternatively, they may represent independent studies into the global range of botulinum neurotoxin-producing bacteria. As such, all available information on isolated strains is desirable, to include the genus/species/lineage of the bacteria, toxin type(s) that may be produced, and the genetic background of these strains.

### 4.4. Food Challenge Studies

Until the late 20th century, foodborne botulism was the most common type of botulism, and its decline was due to improvements in both home and commercial preservation of foods [99,100]. This has required extensive study involving the characteristics surrounding the bacteria that produce these toxins. However, published literature describing botulism outbreaks focuses on the causative strain, with little information on the intrinsic (e.g., pH, salt content, moisture, water activity, addition of antimicrobials) and extrinsic (e.g., packaging, storage temperature) parameters responsible for permitting growth and toxin production of the organism in the food product.

Spores of botulinum neurotoxin-producing clostridia are ubiquitous in the environment and are known contaminants of food commodities and ingredients [101]. Control of these organisms primarily relies on the total destruction of spores within the food product (e.g., retort thermal processing) or through food formulation safety (e.g., adjusting pH, water activity, salt in moisture phase, moisture content, addition of antimicrobials) to prevent the germination of spores and subsequent outgrowth and toxin production within the food product [102]. Knowledge of the growth limitations and physiological characterization of proteolytic and nonproteolytic strains associated with foodborne botulism has advanced our understanding of preventing foodborne botulism outbreaks. Validation of the microbiological safety of foods and food formulations from botulinum neurotoxin production are conducted through inactivation and growth inhibition challenge studies that generally require the use of neuro-toxigenic organisms, which presents a biosafety hazard [103]. Thus, in the United States, these types of studies need to be conducted in special laboratories that are registered with the Federal Select Agent program, and research directed to the use of appropriate nontoxigenic surrogates to enable safer testing options is ongoing [104].

Bacterial spores of proteolytic *C. botulinum* (*C. parabotulinum*) strains demonstrate high heat resistance, which must be eradicated through appropriate temperature and time heat treatment, known as the 12-D “botulinum cook” for retort thermal processing [102]. Regulations involving thermal processing of low-acid canned foods have been established (21 CFR Part 113, “Thermally Processed Low-Acid Foods Packaged in Hermetically Sealed Containers”). Although there are currently no validated surrogates for growth inhibition challenge studies for *C. botulinum*, the non-neuro-toxigenic strain *Clostridium sporogenes* PA3679 is recognized as a validated thermal surrogate for *C. parabotulinum* [105]. Historically, strain PA3679 was considered a *C. sporogenes* strain, but it has recently been determined that certain cultures of PA3679 are nontoxigenic *C. parabotulinum* strains whose spores exhibit high heat resistance, while other cultures labeled as “PA3679” were identified as *C. sporogenes* strains with spores that have a lower heat resistance [106], making them inappropriate as surrogates for this type of testing. This points to the difficulties of using surrogate organisms without determination of their characteristics prior to testing, and the reliance on a species definition to determine the appropriateness of a surrogate organism. This also illustrates the difficulties when the *C. botulinum* species is strictly defined as a toxigenic bacterium.

On the other hand, the ability for nonproteolytic *C. botulinum* bacteria, such as BoNT/E or BoNT/B4 strains, to grow and produce a toxin at refrigeration temperatures poses a different food safety challenge involving vacuum or modified atmosphere packaged (MAP) refrigerated foods [107]. Studies to determine the safety of these foods rely on growth inhibition challenge studies or inoculated pack studies, in which inoculated products are assayed for botulinum neurotoxin production during storage at mild, moderate, or abuse temperatures [103]. Thus, unlike thermal inactivation challenge studies, where nontoxigenic surrogate strains can be used, with these studies the use of nontoxigenic bacteria presents a unique hurdle. A recent effort to replace toxigenic cocktails with nontoxic surrogates [104] involved the identification and characterization of several nontoxic, nonproteolytic strains. Five of these strains were proposed as potential surrogates of nonproteolytic *C. botulinum* in growth inhibition challenge studies based on genomic similarities and appropriate growth characteristics. Side-by-side comparative growth inhibition challenge studies using this nontoxic cocktail versus a toxigenic strain cocktail determined the level of growth of the surrogate cocktail that correlates with the earliest time to toxin production of the toxigenic culture, which is critical in the adoption and implementation of nontoxic surrogates for these studies. This may enable the stringent food safety testing that is necessary to prevent botulism using surrogate organisms in the future, without the precautions needed when using toxigenic bacteria.

A recent study characterized a cocktail composed of ten bacterial strains (seven proteolytic *C. parabotulinum* and three nonproteolytic *C. botulinum*) that are commonly used for growth inhibition challenge studies using whole-genome sequencing and growth competition assays [108]. Overall, this study demonstrated that the strains in this cocktail were representative of the diversity of both *C. parabotulinum* (Group I) and *C. botulinum* (Group II) strains, and although mild competition among strains was observed, robust toxin production was detected in the culture medium when grown together, enabling the use of one cocktail of strains for studies of both proteolytic and nonproteolytic bacteria in a single assay.

As noted above, the choice of appropriate surrogate bacterial strains for use in food safety studies is dependent on the exhibition of appropriate characteristics, i.e., those with spores that are resistant to high heat treatment with thermal inactivation studies, and toxigenic strains that are capable of growth and toxin production under mild- and moderate-temperature abuse conditions for growth inhibition challenge studies. The physiological characteristics are a key factor here, and accurate determination of genus and species designations that reflect these characteristics is necessary when selecting strains for food safety studies. When publishing data from food protection studies, it is important to clearly label your control organisms as to genus, species, toxin type produced, if any, and strain identification, and it is also essential that you have tested your in-house control strains to ensure their identity and applicability for these studies.

### 4.5. Therapeutic BoNT Preparations

Globally, therapeutic botulinum neurotoxin products earn over USD 7 billion dollars annually, making this a very competitive, constantly expanding market [109]. Nomenclature involving therapeutic BoNT preparations is completely different from the serotype/subtype system used in diagnostics or research. The initial therapeutic botulinum neurotoxin products were generally referred to by their brand names or manufacturers. Thus, the name “Botox” is currently probably more famous worldwide than “Xerox”. However, generic names were needed for these toxins when securing FDA approval, so a new vocabulary was developed for distinguishing products that were manufactured by various companies. Table 5 lists some examples of these generic names, brand names, and manufacturers. The generic names do not align with distinct toxin serotypes, subtypes, or even strains. Majority of therapeutic neurotoxin products are produced using BoNT/A1 strains, with the major exception being rimabotulinumtoxinB, which is produced from an unknown toxin subtype of BoNT/B.

While most of these products are produced using the same toxin, strain differences and differences in manufacturing processes result in varying potencies, so it is extremely important to understand the characteristics of the individual toxin product being used for a particular therapeutic or cosmetic application.

Iatrogenic botulism cases (following injection of botulinum neurotoxin), while rare, have occurred, sometimes as a result of overdosing the patient [110]. Clinicians must be acutely aware that they are using the most potent poison known, and they should be thoroughly trained in dosages, injection techniques, and knowledge of their individual product to ensure the application of appropriate toxin doses that will provide maximal effect with minimal side effects.

## 5. Summary

The information presented here is intended to provide an understanding of the complexities involved in the diagnosis of botulism in both human and animal cases, monitoring and research involving food and environmental samples, and the usage of therapeutic botulinum toxin preparations. This complexity can result in difficulty when reporting or researching information related to botulinum neurotoxins and the bacteria that produce them. Accurate, detailed information pertinent to the purpose of the report or article is critical. In clinical work, accurate reporting of the botulinum neurotoxin type is important, while in epidemiology work, comparisons of bacterial isolates are the central factor, and with therapeutic preparations, the generic name for the products is key. Confining terminology to the information which is necessary for a particular purpose can minimize confusion and ensure proper diagnosis, treatment, monitoring of conditions related to both food safety and animal botulism, and application of therapeutic preparations.

## Figures and Tables

**Table 1:** Major discoveries of various toxinotypes with historical and current nomenclature.

Year	Bacterial Species Identifications	Identifications of Associated Toxins	References
1897	First BoNT-producing bacteria isolated (*Bacillus botulinus*) from a nonproteolytic organism	Botulinum toxin verified	[1]
1904	Second *B. botulinus* isolated; different characteristics (proteolytic)	Botulinum toxin verified	[2]
1910, 1919	*B. botulinus*	Discovery that Van Ermengem and Landmann toxins differ serologically, designated type A and type B	[3,4]
1917	*B. botulinus* nomenclature changed to *Clostridium botulinum*		[6]
1922	Proteolytic *C. botulinum* name changed to *C. parabotulinum*	Type C toxin isolated from proteolytic *C. botulinum*, serologically confirmed	[7]
1924	*C. botulinum* name retained to reflect nonproteolytic status	Serologically distinct Type C toxin isolated from nonproteolytic *C. botulinum*	[8]
1928	*C. parabotulinum*	Type D toxin serologically confirmed	[9]
1929	*C. parabotulinum/C. botulinum*	The distinct type C toxins designated Cα, Cβ	[10]
1937	Type E-producing bacterium isolated from nonproteolytic *C. botulinum*	Type E toxin serologically confirmed	[11]
1953	Proposal that all BoNT-producing clostridia be named *C. botulinum*		[12]
1960	Type F-producing bacterium isolated from proteolytic *C. botulinum*	Type F toxin serologically confirmed	[13]
1967	Type F-producing bacterium isolated from nonproteolytic *C. botulinum*	Type F toxin serologically confirmed	[14]
1970	*C. botulinum*	Type G toxin serologically confirmed	[15]
1977	Group system (*C. botulinum* Groups I–IV) instituted for improved detection of BoNT-producing bacteria		[16]
1986	Type E-producing neuro-toxigenic *Clostridium butyricum* bacterium isolated (genetically confirmed)	Type E toxin serologically confirmed	[17]
1988	Type F-producing neuro-toxigenic *Clostridium baratii* bacterium isolated (genetically confirmed)	Type F toxin serologically confirmed	[18]
1988	BoNT/G-producing *C. botulinum* renamed *Clostridium argentinense* due to physiological/genetic differences with *C. botulinum*		[19]
1990	*C. botulinum*	Cβ toxin renamed BoNT/C1 to distinguish from non-neurogenic C2 and C3 toxins that are also present in these strains	[20]
1995	*C. botulinum*	Beginnings of toxin subtype enumerations (A1, A2, etc.)	[21]
1996	*C. botulinum*	Discovery of BoNT/CD (Cα) and BoNT/DC mosaic toxins	[22]
2011	Proposal to rename *C. botulinum* Group III to genospecies *C. novyi sensu lato* due to genomic similarities with *C. novyi*	Produce BoNT/C and BoNT/D toxin subtypes	[23]
2013	*C. botulinum*	Novel toxin identified from an infant botulism case, designated H/FA/HA	[24,25]
2016	Discovery of type B-producing neuro-toxigenic *Clostridium sporogenes*	Produce multiple BoNT/B subtypes	[26,27]
2017	*C. botulinum, Weisella* spp., *Chryseobacterium* spp.	BoNT-like neurotoxins (including BoNT/X) discovered through genomic mining	[28,29,30]

**Table 2:** Botulinum neurotoxin subtypes that are produced by various *Clostridium* species. The same species may produce multiple toxin types and the same toxin type may be produced in multiple species. Species are labeled as described in [36] and physiological group designations are in parentheses.

Toxin Subtypes	Bacterial Metabolic Type	Species/Groups/Lineages
A1–A8, HA	proteolytic	*C. parabotulinum* (*C. botulinum* Group I)
B1–B3, B5–B8	proteolytic	*C. parabotulinum* (*C. botulinum* Group I)
B1, B2, B5, B6	proteolytic	*C. sporogenes* (*C. botulinum* Group I)
B4	nonproteolytic	*C. botulinum* (Group II BEF)
C, CD, D, DC	proteolytic	*C. novyi sensu lato* (*C. botulinum* Group III)
E1–E3, E6–E8, E10–12	nonproteolytic	*C. botulinum* (Group II E)
E4, E5	nonproteolytic	*C. butyricum*
E9	nonproteolytic	*C. botulinum* (Group II BEF)
F1–F5, F8	proteolytic	*C. parabotulinum* (*C. botulinum* Group I)
F6	nonproteolytic	*C. botulinum* (Group II BEF)
F7	nonproteolytic	*C. baratii*
G	proteolytic	*C. argentinense*

**Table 3:** Biophysical and metabolic characteristics of BoNT-producing clostridia. +/− indicates a majority of strains are positive, −/+ indicates a majority of strains are negative, and “weak/variable” indicates reactions are weak or variable. Data are from [52,53]. Columns are labeled with physiological group designations (I–VI) as well as species designations (in parentheses) according to Smith et al. 2018 [36].

	Group I (*C. botulinum*)	Group I (*C. sporogenes*)	Group II (*C. botulinum*)	Group III (*C. botulinum*)	Group IV (*C. argentinense*)	Group V (*C. baratii*)	Group VI (*C. butyricum*)
Lipase produced	+	+	+	+	-	-	-
Lecithinase produced	-	-	-	−/+	-	+	-
Esculin hydrolyzed	+	+	-	-	-	+	+
Starch hydrolyzed	-	-	+/−	-	-	+/−	+
Proteolytic?	yes	yes	no	weak/variable	yes	no	no
Saccharolytic?	no	no	yes	weak/variable	no	yes	yes
Heat resistance of spores	high		low	intermediate			
Optimal growth temperature	35–40 °C	35–40 °C	18–35 °C	40 °C	37 °C	30–45 °C	30–37 °C
Minimal growth temperature	10+ °C		4 °C	15 °C			10 °C

**Table 4:** Susceptible and resistant animals to BoNT toxinotypes. Types that are in parentheses represent rare cases. Information for this table is from Smith and Sugiyama, 1988 [71] and the Merck Veterinary Manual [77].

Laboratory animals	Susceptible to toxin types:
Mice	Susceptible to all toxin types
Guinea pigs	Susceptible to all toxin types
Rats	Resistant to all toxin types except BoNT/A
**Domestic animals**	**Susceptible to toxin types:**
Cattle	C, D, D/C, (A, B, C/D)
Horses	B, C, (A)
Sheep/goats	C, D
Poultry	C/D
Pigs	Appear to be resistant to all toxin types
Minks/foxes/ferrets	C, (A, E)
**Wild animals**	**Susceptible to toxin types:**
Migratory birds	C/D
Waterfowl/fish-eating raptors	C, E
Bighorn sheep	C
Fish	E
Turtles	C
Vultures, flamingos	Appear to be resistant to all toxin types

**Table 5:** A listing of generic names for some therapeutic and cosmetic botulinum neurotoxin products that have applied for FDA approval. Additional information can be obtained through FDA product inserts available on various public websites.

Generic Name	Brand Name(s)	Company	Toxin State
onabotulinumtoxinA	Botox/Botox Cosmetic/Vistabel/Vistabex	Abbvie. Inc. (Allergan), Irving, CA	BoNT/A complex
abobotulinumtoxinA	Dysport/Azzalure	Ipsen (Galderma) Cambridge, MA	BoNT/A complex
incobotulinumtoxinA	Xeomin	Merz Pharmaceuticals Frankfort, Germany	BoNT/A
letibotulinumtoxinA	Botulax/Letybo	Hugel, Inc. South Korea	BoNT/A unknown
nivobotulinumtoxinA	Meditoxin/Botulift	Medytox, Inc South Korea	BoNT/A complex
prabotulinumtoxinA	Jeuveau/Nabota	Daewoong Pharmaceutical South Korea	BoNT/A complex
daxibotulinumtoxinA-lanm	Daxxify	Revance Therapeutics Nashville, TN	BoNT/A
rimabotulinumtoxinB	MyoBloc/Neurobloc	Solstice Neurosciences, LLC South San Francisco, CA	BoNT/B complex
